# Prevalence and factors associated with irregular red blood cell antibodies among blood donors in Eastern Tanzania

**DOI:** 10.1371/journal.pone.0362471

**Published:** 2026-11-12

**Authors:** Mariam Charles Monah, Vulstan James Shedura, Peter Richard Torokaa, Loveness Urio, Seif Abdul, Rosemary Bulemo, Mariam R. Mbwana, Sephord S. Ntibabara, Oscar Mwashiuya, Beatrice Tingo, Macdonald Mahiti, Upendo Kibwana, Salim Masoud, Joel Manyahi, Mtebe Majigo, Agricola Joachim, Doreen Kamori

**Affiliations:** 1 Muhimbili University of Health and Allied Sciences, Dar es Salaam, Tanzania; 2 Tanzania Field Epidemiology and Laboratory Training Programme, Dar es Salaam, Tanzania; 3 Ministry of Health, Southern Zone Referral Hospital, Mtwara, Tanzania; 4 National Public Health Laboratory, Dar es Salaam, Tanzania; 5 Eastern Zone Blood Transfusion Centre, Dar es Salaam, Tanzania; 6 National Blood Transfusion Service, Dar es salaam, Tanzania; 7 Northern Zone Blood Transfusion Centre, Kilimanjaro, Tanzania; Australian Red Cross Lifeblood, AUSTRALIA

## Abstract

**Background:**

Irregular red blood cell (RBC) antibodies are antibodies other than naturally occurring anti-A and anti-B. Although the global prevalence of these antibodies is relatively low, ranging from 0.2% to 2%, donors who have irregular RBC antibodies can present immunological risk for patients due to hemolytic transfusion reactions. Hence, it is imperative to determine the prevalence of these antibodies in resource-limited settings where laboratory infrastructure for screening is limited. Therefore, this study aimed to determine the prevalence and factors associated with irregular RBC antibodies among blood donors in Dar es Salaam, Tanzania.

**Methods:**

A cross-sectional study was conducted from 01^st^ April to 31^st^ May 2024. Random sampling was used to select participants. The data were summarized by using frequency and percentage for categorical variables and mean and standard deviation for continuous variables. The logistic regression model was used to assess the relationship between the presence of irregular RBC antibodies and the independent variables. A *p*-value of < 0.05 was considered significant.

**Results:**

A total of 501 blood donors were enrolled in the study. The participants had a mean age of 32 years and a standard deviation (SD) of 9.48. The majority, 65.3% of participants, were male, and 83.6% had no history of blood transfusion. The overall prevalence of irregular RBC antibodies was 7.6% (95% CI 5.5%−10.3%). Being female (adjusted odds ratio [aOR] 3.4, 95% CI 1.72–6.85), age over 40 years (aOR 4.4, 95% CI 1.29–15.00), and having a history of blood transfusion (aOR 3.1, 95% CI 1.53–6.42) were significantly associated with irregular RBC antibodies.

**Conclusion:**

The present study revealed a relatively high prevalence of irregular RBC antibodies among eligible blood donors in Tanzania. Therefore, these findings highlight the need for enhanced practices in screening and the prudent use of blood and blood products in Tanzania.

## Introduction

Irregular red blood cell (RBC) antibodies are antibodies other than naturally occurring anti-A and anti-B antibodies on RBCs [1]. The irregular RBC antibodies may be present in plasma from blood donors. They can lead to transfusion reactions in adult and pediatric recipients who receive multiple transfusions [2], however the haemolytic transfusion reactions are not only a risk for paediatric patients.

The development of irregular RBC antibodies in healthy donors is primarily stimulated by prior blood transfusions or pregnancies, with key factors associated with alloimmunization including female sex (linked to pregnancy-related exposure), previous transfusions, and sickle cell disease, as well as variations in blood group compatibility, ethnicity, and healthcare practices across different regions [3,4]. These antibodies are classified as alloantibodies, formed in response to foreign RBC antigens, and autoantibodies, which react against self-antigens often associated with autoimmune conditions. Clinically significant alloantibodies include Anti-D, Anti-K, Anti-E, and others that target antigens from the Rhesus (Rh), Kell, Kidd, MNS, and Duffy blood group systems. In contrast, autoantibodies such as Anti-e (warm) and Anti-I (cold) are typically linked to the Rh or I systems [5,6].

Globally, the prevalence of irregular RBC antibodies ranges from 0.2% to 2% among blood donors, with regional variations depending on population characteristics and laboratory methods [7–9]. Studies in India, Saudi Arabia, and China have reported a prevalence ranging from 0.27% to 3% among whole-blood donors [8,10]. In South America, a Brazilian study identified irregular RBC antibodies in 0.4% of blood donations, alongside a 2.6% prevalence of abnormal hemoglobin [11].

In Sub-Saharan Africa, the prevalence of irregular RBC antibodies varies widely, ranging from 0.14% to 7.4%, with higher rates reported in multi-transfused populations, such as patients with sickle cell disease, where the prevalence can reach 28% [3,4]. In East Africa, data on irregular RBC antibodies remain limited; however, a study conducted at the central blood bank laboratory in Nairobi, Kenya, reported a prevalence of 4% among blood donors [12]. Despite routine antibody screening tests being performed at National Blood Transfusion Centers in Tanzania, there is limited information on the prevalence and factors associated with irregular RBC antibodies among blood donors. Therefore, the present study aimed to determine the prevalence and factors associated with irregular RBC antibodies at the Eastern Zone Blood Transfusion Center (EZBTC) in Dar es Salaam, which contributed to 1.8% (10,514/578,827) of the blood units donated in the country.

## Materials and methods

### Study design and settings

This cross-sectional study was conducted from 01^st^ April 2024–31^st^ May 2024 at the Eastern Zone Blood Transfusion Center located in Dar es Salaam. The center collects approximately 10,000 units of blood each year from voluntary donors. Most donations come from three regions Dar es Salaam, Pwani, and Morogoro as well as from walk‑in donors at the zonal blood transfusion center. The zonal team, in collaboration with hospital teams, collects blood in different areas in Dar es Salaam and other regions within the zone. Collected blood serves three regions; Morogoro, Dar es Salaam, and Pwani, as well as any other region in the country that may require blood. Participants in this study included walk‑in donors at the EZBTC and donors at selected mobile collection sites. Mobile sites were purposefully selected due to their large population density, including Buguruni Bus Stand, Mbagala Rangi Tatu Market, Sinza Mori, and Ilala Market.

### Study population

The study population consisted of voluntary non-remunerated blood donors, who were considered to be in a safer category for blood donation. Both male and female blood donors aged between 18 and 65 years who donated blood from 01^st^ April 2024–31^st^ May 2024 in Dar es Salaam were included in the study. Blood donors who had a history of autoimmune conditions were excluded from the study.

### Sample size estimation and sampling method

The sample size was calculated using a single-proportion formula based on data from a prevalence study conducted in Cameroon, which reported a prevalence of irregular RBC antibodies of 5.0% [9]. The 95% confidence interval (CI), a marginal error of 2%, and considering a 10% non-response rate. The resulting minimum sample size was 501 participants. Random sampling was used to select participants from different mobile sites during blood collection.

### Data collection

We used a well-structured questionnaire to capture demographic information (age, sex, and residence) and clinical details (history of blood transfusion). Furthermore, we collected a detailed gynecological and obstetrical history from the female blood donors. The questionnaire was administered by trained research assistants.

### Laboratory procedures

Blood samples for the antibody test were collected from donors in an EDTA (Ethylene Diaminetetraacetic Acid) tube. Antibody screening from blood donors’ samples was performed using the microplate method with commercially available pooled O cells (Capture-R ready screened cells) on a fully automated machine (NEO Iris fully automated blood bank serological analyzer, 6^th^ generation, Werfen, United Kingdom, 2023). Briefly, the microplates contained dried polystyrene micro wells which have been coated with RBC membranes. The membrane antigens are used to capture RBC specific antibodies from donor plasma. Following an incubation period at 37 °C, unbound residual immunoglobulins were rinsed from the wells and replaced with a suspension of anti-IgG-coated indicator RBCs (Capture-R Ready Indicator Red cells). The centrifugation process, which took place in 3 minutes at 500RCF (Relative Centrifugal Force), was done by bringing indicator red cells in contact with antibodies bound to the RBC membrane. Results were reported qualitatively, either positive or negative. Positive results indicate the presence of irregular antibodies in donated blood, and negative results indicate the absence of irregular antibodies in donated blood.

### Data analysis

Data were extracted from the Kobo Collect tool, uploaded, and cleaned using Microsoft Excel 2022. The data were uploaded and analyzed using STATA version 15 (StataCorp, 2017). The data were summarized by using frequency and percentage for categorical variables and mean and standard deviation (SD) for continuous variables. Logistic regression analysis was used to determine factors associated with irregular RBC antibodies among blood donors. Factors with a p-value of 0.20 in the bivariate analyses were included in the multivariable logistic regression model. The 95% confidence interval (CI) was presented to assess the strength of association, and a *p*-value of < 0.05 was considered significant.

### Ethical consideration

Ethical approval was obtained from the Senate Research and Publications Committee (SRPC) of Muhimbili University of Health and Allied Sciences (MUHAS) (Ref. No. MUHAS-REC-02-2024-2033). Approval to collect data from blood donors was obtained from the National Blood Transfusion Service Program Manager in the Ministry of Health (MoH). All the participants provided informed written consent. The participant’s information was strictly dealt with confidentiality.

## Results

### Demographic and clinical characteristics of study participants

A total of 501 blood donors participated in this study (S1 File). The mean age of donors was 32 years, with a SD of 9.48. The majority of the participants, 265/501 (52.9%), were aged between 25 and 40 years. The majority, 327/501 (65.3%) of the participants were males, and 399/501 (79.6%) resided in urban areas of the Dar es Salaam region. Only 82/501 (16.4%) had a history of blood transfusion (Table 1).

**Table 1:** Distribution of irregular RBC antibodies among blood donors at Eastern Zone Blood Transfusion Center (EZBTC) by demographic and clinical characteristics (N = 501).

Characteristics	Frequency n (%)	Irregular antibodies		*p value*
		Negative n (%)	Positive n (%)	
**Age (years)**				
18-24	140 (27.9)	136 (97.1)	4 (2.9)	
25-40	265 (52.9)	244 (92.1)	21 (7.9)	
>40	96 (19.2)	83 (86.5)	13 (13.5)	**0.009**
**Sex**				
Male	327 (65.3)	311 (95.1)	16(4.9)	
Female	174 (34.7)	152 (87.4)	22(12.6)	**0.002**
**Residence**				
Rural	102 (20.4)	96 (94.1)	6 (5.9)	
Urban	399 (79.6)	367 (92.0)	32 (8.0)	0.467
**Education level**				
Primary	141 (28.1)	126 (89.4)	15 (10.6)	
Secondary	200 (40.0)	186 (93.0)	14 (7.0)	
College & University	160 (31.9)	151 (94.4)	9 (5.6)	0.241
**History of Blood Transfusion**				
Yes	82 (16.4)	67 (81.7)	15 (18.3)	
No	419 (83.6)	396 (94.5)	23 (5.5)	**<0.001**
**Number units Transfused**				
One	43 (52.4)	38 (83.4)	5 (11.6)	
More than one	39 (47.6)	29 (74.4)	10 (25.6)	**<0.001**
Duration of last Blood Transfusion*				
≤ 1 year	12 (14.6)	11 (91.7)	1 (8.3)	
> 1 year	70 (85.4)	56 (80.0)	14 (20.0)	<0.001
History of Pregnancy*				
Yes	111 (63.8)	92 (82.9)	19 (17.1)	
No	63 (36.2)	60 (95.2)	3 (4.8)	**<0.001**
Parity*				
One	35 (31.5)	30 (85.7)	5 (14.3)	
More than one	76 (68.5)	62 (81.6)	14 (18.4)	0.591

*Indicates the number of participants involved in the analysis is < 501.

### Prevalence of irregular RBC antibodies among blood donors

The overall prevalence of irregular RBC antibodies among blood donors was 7.6% (38/501), with a (95% CI: 5.5–10.3%). A higher proportion of irregular RBC antibodies was observed in individuals aged 40 years and older compared to other age groups (*p* = 0.009). Female participants had a significantly higher proportion of irregular RBC antibodies compared to male participants (*p* = 0.002). Individuals with a history of blood transfusion had a significantly higher proportion compared to those with no history of blood transfusion (*p* < 0.001). Individuals with a history of pregnancy showed a higher proportion of irregular RBC antibodies than those without a history of pregnancy (*p* = 0.018) (Table 1).

### Factors associated with irregular RBC antibodies among blood donors

Our findings showed that blood donors aged greater than 40 years had 4.4 times the odds of having irregular RBC antibodies compared to blood donors aged between 18 and 24 years [aOR 4.4, 95%CI: 1.29–15.00, *p* = 0.018]. Being female was independently associated with irregular RBC antibodies, where female donors were 3.1 times more likely to have irregular RBC antibodies compared to male donors [aOR 3.1, 95% CI: 1.49–6.55, *p* = 0.002]. Blood donors who had a history of previously being transfused with blood have 2.6 times the odds of having irregular RBC antibodies [aOR 2.6, 95% CI: 1.20–5.62, *p* = 0.015] compared to those who did not have a history of blood transfusion. In the present study, the other factors, including the place of residence and education level, were not significantly associated with Irregular RBC antibodies among blood donors at Eastern Zone Blood Transfusion Centre (EZBTC) (Table 2).

**Table 2:** Bivariate and Multivariate logistic regression on factors associated with irregular RBC antibodies among blood donors at Eastern Zone Blood Transfusion center (EZBTC).

Characteristics	Irregular antibodies n (%)	Crude Odds Ratio		Adjusted Odds Ratio	
		cOR (95% CI)	*p-*value	aOR (95%CI)	*p*- value
**Age (in years)**					
18-24	4 (2.9)	1		1	
25-40	21 (7.9)	2.9 (0.98-8.70)	0.053	2.7 (0.87-8.24)	0.087
>40	13 (13.5)	5.3 (1.68-16.88)	0.004	4.4 (1.29-15.00)	**0.018**
**Sex**					
Male	16(4.9)	1		1	
Female	22(12.6)	2.8 (1.43-5.51)	0.003	3.1 (1.49-6.55)	**0.002**
**Residence**					
Rural	6 (5.9)	1		1	
Urban	32 (8.0)	1.4 (0.56-3.43)	0.469	–	–
**Education level**					
Primary	15 (10.6)	2.0 (0.84-4.71)	0.115	–	–
Secondary	14 (7.0)	1.3 (0.53-2.99)	0.597	–	–
College & University	9 (5.6)	1		1	
**History of Blood Transfusion**					
Yes	15 (18.3)	3.8 (1.91-7.76)	< 0.001	2.6 (1.20-5.62)	**0.015**
No	23 (5.5)	1		1	
**Frequency of Blood Transfusion**					
One Unit	5 (11.6)	1		1	
More than one unit	10 (25.6)	2.6 (0.81-8.51)	0.109	–	–
**Duration of Blood Transfusion**					
≤1 year	1 (8.3)	1		1	
>1 year	14 (20.0)	2.8 (0.33-23.12)	0.352		–
**History of Pregnancy**					
Yes	19 (17.1)	4.1 (1.12-14.57)	0.029	–	–
No	3 (4.8)	1		1	
**Parity**					
One	5 (14.3)	1		1	
More than one	14 (18.4)	1.4 (0.44-4.11)	0.592	–	–

cOR- Crude odds ratio, aOR- Adjusted odds ratio, Ref- Reference group, CI-Confidence interval.

In the multivariable model, the variables frequency of blood transfusion, duration of blood transfusion, history of pregnancy, and parity were excluded due to sparse data and estimation problems. As a result, the model could not generate stable estimates for these independent variables.

## Discussion

In the present study, the prevalence of irregular RBC antibodies among blood donors in Eastern Tanzania was found to be relatively high, indicating a significant immunohematological concern in transfusion safety. This prevalence aligns with findings from studies conducted in Kenya and Cameroon, which reported prevalence rates of 4–5%, suggesting a comparable burden within sub-Saharan Africa, where similar screening methodologies are employed [9,13]. However, the prevalence observed in the present study was markedly higher than that reported in India (0.3%), which may be attributed to population differences, genetic variability in antigen expression, and disparities in laboratory screening techniques [14].

The relatively high prevalence in Tanzania underscores the need for strengthened antibody screening protocols, particularly in blood transfusion centers, to minimize the risk of hemolytic transfusion reactions. Clinically, these findings highlight the importance of routine screening for irregular antibodies prior to transfusion, especially among female donors, older adults, and individuals with a previous history of transfusion, who were identified as high-risk groups in this study. Among donors younger than 24 years, 8 reported a previous blood transfusion, and 11 reported a history of pregnancy. However, none of the participants in this age group who were alloimmunized had a history of either blood transfusion or pregnancy.

The present study found that female donors were 3.1 times more likely to have irregular RBC antibodies compared to males. The high prevalence of irregular RBC antibodies observed among female donors in our study aligns with findings from a study in India, which reported a prevalence of 4.5% among female donors [14]. Other studies highlight pregnancy as a major immunizing event predisposing women to alloimmunization [8,14]. The exposure to antigens during pregnancy may be the cause of this trend [15]. During pregnancy, fetal-maternal hemorrhage exposes women to paternal antigens, leading to antibody formation that that may persist in future blood donations. Such discrepancies may be attributed to differences in donor demographics, transfusion histories, and study methodologies [10]. However, contrasting findings have been reported in other studies in India, where male donors showed higher prevalence rates of 84.2% and 82.9%, respectively [8,16]. Lower antibody prevalence in females than in males likely reflects population differences, transfusion histories, or parity rates [3].

The prevalence of irregular RBC antibodies was higher among donors with a history of blood transfusion compared to those who had never been transfused. These results are comparable to those reported in a study conducted in Zambia, which reported a prevalence of 6% of irregular RBC antibodies among individuals with a history of blood transfusion [17]. Our finding is also consistent with those from an extensive multi-center study in the United States, where previously transfused donors were nearly four times more likely to have alloantibodies than non-transfused donors [8]. Repeated transfusions expose individuals to a broader range of foreign antigens, thereby increasing the risk of alloimmunization. However, variations across studies may stem from differences in screening methods, antigenic diversity of donor pools, and transfusion practices in different regions.

In this study, we report that irregular RBC antibodies are independently associated with blood donors aged 40 years or older. These findings are consistent with those of another study, which also demonstrated an increase in antibody screen positivity with increasing age [18,19]. Beyond cumulative exposure to transfusions, pregnancies, or hemorrhagic events, age-related immune changes (immunosenescence) and repeated antigenic stimulation may have also contributed to higher alloimmunization rates in older donors, as shown in a previous study [20].

There was no statistically significant relationship between parity and irregular RBC antibodies in this study. This observation is similar to a study conducted in Egypt, which showed no significant association between parity in healthy blood donors, and irregular RBC antibodies [21]. In contrast, a study in the eastern region of India showed a significant association between parity status and the formation of irregular RBC antibodies [22,23]. This discrepancy can be influenced by different approaches to manage pregnancy-related health issues, such as prenatal care, managing pregnancy-related problems, and childbirth interventions, which can affect the immune responses of mothers and the subsequent development of irregular antibodies.

The study has several limitations. Firstly, only gravidity was recorded, with miscarriages not captured despite their potential to elicit alloimmunization. Although the intention was to document gravidity, most likely the participants only really thought to disclose parity/may have had pregnancy they were unaware of. Secondly, testing was not performed to determine whether donors had autoantibodies, meaning that some positive screens may have reflected autoantibodies rather than alloantibodies. Thirdly, the specificity of alloantibodies was not established, as reactivity was assessed only with pooled cells and donor blood groups such as RhD were not reported. Fourthly, detailed information on national transfusion policies and practices was lacking, including whether transfusions were predominantly whole blood or component-based. Fifthly, no data were collected on whether recipients were routinely screened for antibodies or consistently cross-matched. Sixthly, the study did not document whether pregnant women were systematically screened for alloantibodies, followed by maternal monitoring and neonatal screening for jaundice. Sevenly, transfusion history was self-reported, including the number of transfusions received, which may have led to under-reporting for example, participants recalling only a single transfusion when multiple may have occurred. Such recall bias could have influenced the findings.

A key strength of this study lies in its large and representative sample of blood donors, encompassing diverse age groups, both sexes, and varying educational and residential backgrounds, thereby enhancing the generalizability of the findings to the donor population in Eastern Tanzania.

## Conclusion

The present study revealed a high prevalence of irregular RBC antibodies among blood donors. Having older age (>40 years), being female, and having a history of blood transfusion are factors independently associated with irregular RBC antibodies among blood donors in Eastern Tanzania. These findings underscore the need to refine guidelines on the safe use of blood and blood products and strengthen immunohematology services and capacity-building in transfusion laboratories, aiming to reduce transfusion reactions, enhance transfusion safety, and reduce transfusion-related adverse outcomes in Tanzania and similar settings.

## Supporting information

S1 File Irregular antibodies dataset. (XLSX)
